# One-step partial or complete caries removal and bonding with antibacterial or traditional self-etch adhesives: study protocol for a randomized controlled trial

**DOI:** 10.1186/s13063-016-1484-0

**Published:** 2016-08-15

**Authors:** Cyril Villat, Jean-Pierre Attal, Nathalie Brulat, Franck Decup, Sophie Doméjean, Elisabeth Dursun, Hélène Fron-Chabouis, Bruno Jacquot, Michèle Muller Bolla, Nelly Plasse-Pradelle, Laurent Roche, Delphine Maucort-Boulch, Patrice Nony, Kerstin Gritsch, Pierre Millet, François Gueyffier, Brigitte Grosgogeat

**Affiliations:** 1 Université Lyon 1 and Hospices Civils de Lyon, LMI UMR CNRS, 5615 Lyon, France; 2 Université Paris Descartes and Assistance Publique des Hôpitaux de Paris, URB2i, EA 4462 Paris, France; 3 Université de Nice Sophia Antipolis and CHU de Nice, Mines Paris Tech, CEMEF, UMR, CNRS 7635 Nice, France; 4 Université Paris Descartes and Assistance Publique des Hôpitaux de Paris, EA 2496 Paris, France; 5 Université d’Auvergne Clermont-Ferrand and CHU de Clermont-Ferrand, CROC, EA 4847 Clermont-Ferrand, France; 6 Université d’Aix-Marseille and Assistance Publique des Hôpitaux de Marseille, BioSanté, EA 4203 Marseille, France; 7 Université de Nice Sophia Antipolis and CHU de Nice, URB2i, EA 4462 Nice, France; 8 Université Paris Diderot and Assistance Publique des Hôpitaux de Paris, LMI UMR CNRS, 5615 Paris, France; 9 Université Lyon 1 and Hospices Civils de Lyon, LBBE UMR CNRS, 5558 Lyon, France; 10 Université de Reims Champagne Ardenne and CHU de Reims, LISM, EA4695 Reims, France; 11 Université Lyon 1. UFR d’Odontologie, 11 rue Guillaume Paradin, 69372 Lyon Cedex 08, France

**Keywords:** Deep carious lesion, Cavity preparation, Partial caries removal, Permanent dentition, Dental adhesives, Composite resins, Pulp capping, Randomized controlled trial

## Abstract

**Background:**

Current concepts in conservative dentistry advocate minimally invasive dentistry and pulp vitality preservation. Moreover, complete removal of carious dentin in deep carious lesions often leads to pulp exposure and root canal treatment, despite the absence of irreversible pulp inflammation. For years, partial caries removal has been performed on primary teeth, but little evidence supports its effectiveness for permanent teeth. Furthermore, the recent development of new antibacterial adhesive systems could be interesting in the treatment of such lesions. The objectives of this study are to compare the effectiveness of partial versus complete carious dentin removal in deep lesions (primary objective) and the use of an antibacterial versus a traditional two-step self-etch adhesive system (main secondary objective).

**Methods/Design:**

The DEep CAries Treatment (DECAT) study protocol is a multicenter, randomized, controlled superiority trial comparing partial versus complete caries removal followed by adhesive restoration. The minimum sample size required is 464 patients. Two successive randomizations will be performed (allocation ratio 1:1): the first for the type of excavation (partial versus complete) and the second (if no root canal treatment is required) for the type of adhesive (antibacterial versus traditional). For the two objectives, the outcome is the success of the treatment after 1 year, measured according to a composite outcome of five FDI criteria: material fracture and retention, marginal adaptation, radiographic examination (including apical pathologies), postoperative sensitivity and tooth vitality, and carious lesion recurrence.

**Discussion:**

The study will investigate the interest of a conservative approach for the management of deep carious lesions in terms of dentin excavation and bioactive adhesive systems. The results may help practitioners achieve the most efficient restorative procedure to maintain pulp vitality and increase the restoration longevity.

**Trial registration:**

ClinicalTrials.gov Identifier NCT02286388. Registered in November 2014.

**Electronic supplementary material:**

The online version of this article (doi:10.1186/s13063-016-1484-0) contains supplementary material, which is available to authorized users.

## Background

Currently, caries management should be based on minimal intervention dentistry concepts [1], including minimally invasive cavity preparations when surgical intervention is required. However, with deep carious lesions, the most commonly used procedure is complete excavation in one step, with hardness to a dental probe used to assess caries excavation [2, 3]. This practice leads to a high risk of pulp exposure and poor prognosis in terms of maintaining pulp vitality [4].

Two alternative options have been proposed to minimize the risks of pulp exposure and postoperative pulpal complications.

The first strategy, chronologically, is the stepwise technique (complete excavation in two steps), with residual carious dentin at the pulpal wall of the prepared cavity left under a temporary restoration placed at the first visit followed by a complete excavation at a second visit (generally 2–6 months later). Clinical surveys have compared the stepwise technique with one-step complete excavation in permanent teeth [5, 6], primary teeth [7], and mixed dentition [8]. Nevertheless, the stepwise technique is time-consuming and costly, and pulp exposure occurs in 15–20 % of cases during the second excavation step [9].

The second approach, more controversial due to a paradigm shift, is partial (incomplete/selective) excavation. The clinical procedure is the same as with the stepwise excavation but without any second procedure. To avoid pulp exposure, carious dentin remains close to the pulp and the cavity is sealed with a definitive restoration. This concept is based on substantial evidence that removal of all carious dentin in deep carious lesions is not required for successful lesion management, provided that the restoration can effectively seal the lesion from the oral environment [10]. Studies have compared stepwise with partial excavation techniques only in permanent teeth [11, 12] or complete excavation in one step with partial excavation essentially in primary teeth [13–17] and mixed dentition [8]. Partial excavation results in lower long-term costs, longer-retained teeth and better pulp preservation than complete excavation (in one or two steps). However, because of high risk of bias within studies, more randomized clinical trials are needed for definitive conclusions [9, 18, 19].

Two multicenter randomized clinical trials are currently ongoing, one comparing selective and stepwise excavation of deep carious lesions in primary molars [20] and our study comparing complete excavation in one step to partial excavation in permanent teeth.

Furthermore, after dentine excavation, different materials may be used to restore the loss of tooth substance [21]. Glass ionomer cements (GIC) are commonly used because of their biocompatibility, fluoride release and adhesive properties, but they have weak mechanical and aesthetic properties [22]. Dental composite resins have high mechanical and good aesthetic properties but limited bioactive properties [23] and require a specific adhesive system. Few clinical studies have evaluated the role of antimicrobial agents incorporated into restorative materials and their potential anti-caries effect [24]. An effective bactericidal effect from adhesive systems could be an alternative to suppress residual contamination after caries removal [25], even more so with incomplete eviction. Adhesive systems usually have limited bioactivity. A new adhesive, Clearfil™ SE Protect (Kuraray Europe Gmbh, Hattersheim, Germany), is the only adhesive system considered to have antibacterial properties. This adhesive system contains 12-methacryloyloxydodecylpyridinium bromide (MDPB), which inhibits the proliferation of bacteria involved in dentinal caries like *Streptococcus mutans*, *Lactobacillus casei* and *Actinomyces naeslundii* and reduces lactic acid production of *S. mutans* [26–30]. Moreover, it has been shown that, in vivo, this adhesive system can induce the inhibition of caries progression [31] and preservation of pulp vitality [32].

However, no clinical study has evaluated this type of adhesive for conservative treatment of deep carious lesions.

### Objectives and hypotheses

The aim of this clinical trial is to support a management strategy that preserves pulp vitality in cases of deep carious lesions with partial caries removal, followed by a composite restoration, in a single session.

The primary objective is the comparison of the efficacy (binary success criteria) of partial (P) versus complete (C) excavation in deep carious lesions of mature permanent teeth at 1 year. The main secondary objective is the efficacy (binary success criteria) of an antibacterial adhesive (AA) versus a traditional two-step self-etch adhesive (TA) at 1 year. Two secondary objectives will be also considered: (i) the identification of predictive factors for success at 1, 2, and 3 years and (ii) the description of the consequences of treatment failure and side effects for each treatment arm.

The underlying pathophysiological hypotheses are that (1) partial excavation would imply less irreversible pulp diseases as compared with complete excavation in one step and (2) the antibacterial adhesive system would help prevent secondary caries.

## Methods/design

### Trial design

The Standard Protocol Items: Recommendations for Interventional Trials (SPIRIT) guidelines [33] have strictly been followed in the planning of this trial (Additional file 1).

This trial is a multicenter intention-to-treat randomized controlled superiority trial with parallel arms. Analysis of results will be performed according to the intention-to-treat principle. For the primary objective, only patients are blinded to the allocated intervention (partial or complete caries removal). For the secondary objective (type of adhesive), both patients and investigators are blinded. Outcome examiners and data analysts are always blinded.

Two successive randomizations are performed (allocation ratio 1:1): first, for the type of excavation (P versus C) and second, for the adhesive system (AA) versus TA. The second randomization is not performed for teeth requiring endodontic treatment after the first randomization.

**Fig. 1:**
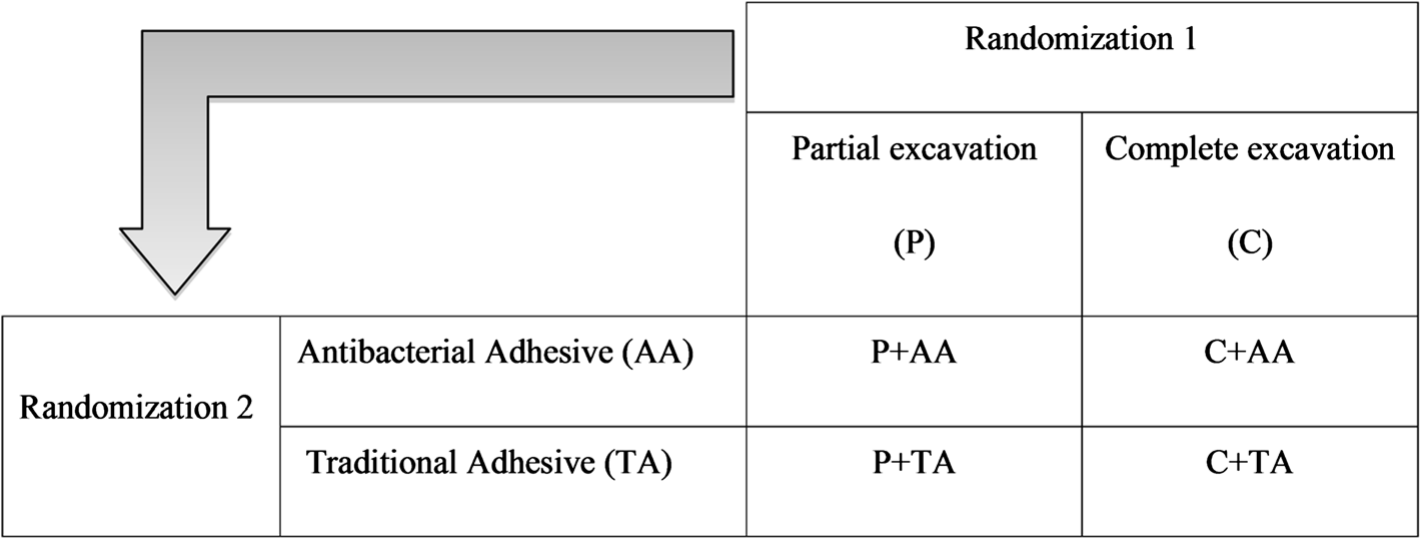
Combinations of interventions

**Fig. 2:**
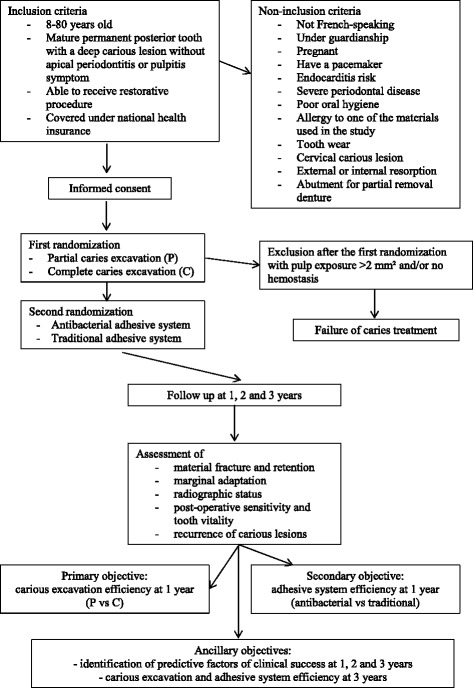
Flow chart

The possible combinations of interventions are in Fig. 1, the flow diagram is mentioned on Table 1 and the flow chart of the study is in Fig. 2.

**Table 1:** Flow diagram

	Study period					
	Selection visit	Randomization visit	Postrandomization visit			Emergency visit
Time point	T-1	T0	T0 + 1 yr	T0 + 2 yrs	T0 + 3 yrs	Between T0 and T0 + 3 yrs
Enrolment:						
- eligibility screen	X					
- informed consent	X					
- randomization		X				
Interventions:						
- caries management		X				If needed
- X-ray	X	X	X	X	X	If needed
Control of carious eviction with the SoproLife*®*		X				
assessment:						
- baseline variables	X	X				
- FDI criteria			X	X	X	X
- other data variables listed in the CRF			X	X	X	X

*FDI* FDI World Dental Federation, *CRF* case report form

### Setting and participants

This study is being carried out in 13 academic hospitals and two private practices in France (study sites can be found at ClinicalTrials.gov; inclusion from February 2015 to July 2016).

Eligible patients, and their parent(s), if necessary, receive the study information. Informed consent forms are given to the patient and to the parent(s) of minor patients (age 8–18 years), before inclusion.

To be included in the trial, patients must 8–80 years old, able to receive dental care, covered under the national health coverage system, and have a mature permanent posterior tooth affected by a deep carious lesion. The tooth should have normal response to pulp sensitivity tests, without periapical pathology, and require direct restorative procedure. Exclusion criteria are not French-speaking, under guardianship or pregnant, have a pacemaker, or at risk of infectious endocarditis. Other exclusion criteria are severe periodontal disease (axial or high lateral mobility and/or pocket depth more than 5 mm), poor oral hygiene (patient with abundant plaque, i.e., plaque index = 3 according Silness and Loe plaque index) and allergy to any component of the materials used in the procedure. Teeth with excessive wear, carious cervical lesion, external or internal resorptions, or abutment for removable partial denture are not eligible. Exclusion criteria have been chosen to reduce loss of follow-up or modifications independent of the treatments studied.

Medications (painkillers, non-steroidal anti-inflammatory drugs) taken less than 8 days before or during the study must be reported.

No special concomitant care or intervention is prohibited after inclusion in the trial, except that concerning the included tooth.

### Sample size

According to a literature review [9, 34–42], 97 % and 90 % of success is expected for P and C dentin excavation strategies, respectively. A total of at least 464 patients would be needed for a bilateral test with an alpha risk of 5 % and a power greater than 80 %, supposing at 1 year 10 % of patients lost to follow-up as a ‘not to exceed’ value (calculated by nQuery Advisor 7.0) [43–45].

With the expected recruitment of each investigator, in order to reach the target sample size and respect the inclusion period of 18 months, this trial involves 15 centers. To encourage recruitment, the main investigator sends an encouraging text message after each inclusion and the center recruitment appears on the main page of the randomization website.

### Randomization

Randomization is performed using a computerized and centralized system via a specific website. A two-stage randomization process allows for integrating the two levels of randomization (i.e., the type of excavation and the nature of the adhesive). A minimization procedure is implemented to take into account the following prognostic characteristics: investigator, patient age (by age group), type of carious lesion (primary or secondary), location of the lesion (proximal or occlusal) and pulp exposure.

Because all patient and cavity characteristics must be entered in the electronic case report form (eCRF) before interventions are assigned, sequence concealment is secured.

### Implementation

The minimization algorithm was implemented by statisticians and methodologists independently of investigators. Patients are assigned to interventions arm by the investigators.

### Intervention

At the first visit, eligibility criteria are verified by an investigator. Information about the study is then given to the patient and the inclusion is formalized by the signing of the informed consent form.

A clinical examination is performed, then (1) pulp vitality is assessed with thermal (cold) and electrical methods (Elements Diagnostic Unit and Apex Locator®, Kerr Endodontique, Bioggio, Switzerland); and (2) bite-wing and retroalveolar radiographs are taken by using film-holders supplied by Kerr Endodontique. Bite-wing radiographs allow for assessing the depth of the lesion: no continuity between the carious lesion and the pulp chamber should be seen; and retroalveolar radiographs allow for determining the periapical index [46]. In case of multiple carious lesions, the lesion that best meets the inclusion criteria and allows for the best handling (easy access) is chosen. Other lesions will be further treated according to usual procedures and will not be entered in the study.

At the second visit, an investigator performs the tooth restoration. The tooth is anesthetized. The shade of the tooth-colored restoration is selected before isolation with the rubber dam. The first randomization is achieved. The superficial necrotic tissues and peripheral demineralized dentin are removed with complete excavation to avoid excavation close to the pulp. The bulk of the carious dentin is removed by using sterile round ceramic burs (Komet France, Paris, France) under water spray cooling and the final excavation with hand instruments. The excavation is controlled by use of an intraoral light-induced fluorescence camera (Soprolife®, Acteon Group, La Ciotat, France) and the corresponding image is registered. According to the manufacturer of the Soprolife® camera, infected carious tissues appear green-black in fluorescence. The infected/affected dentin interface emits bright red fluorescence in active lesions and dark red fluorescence in arrested lesions. The underlying dentin looks gray-green in fluorescence [47].

For cases under P excavation, the investigator stops excavating when the dentin close to the pulp appears bright or dark red after removal of the green-black infected dentin.

For cases under C excavation, the investigator stops excavating when the dentin close to the pulp appears gray-green after removal of the green-black infected dentin and removal of the bright or dark red infected/affected dentin interface.

With pulp exposure < 2 mm^2^ after C excavation, the exposed pulp is irrigated with chlorhexidine. Following hemostasis (within 5 minutes), a tricalcium silicate cement (Biodentine™, Septodont, Saint Maur des Fossées, France) is applied according to the manufacturer’s instructions. After complete cement setting (within 15 minutes), the cavity is restored following the same clinical procedures as for cases without pulp exposure.

With pulp exposure > 2 mm^2^ and/or no hemostasis, a root canal treatment is performed, and the case is considered a failure for randomization 1 but not randomization 2.

Cases undergo randomization 2 to determine the adhesive system: Clearfil™ SE Bond (Kuraray Europe) or Clearfil™ SE Protect (Kuraray Europe) (i.e., TA and AA, respectively). Enamel and dentin are prepared according to the manufacturer’s instructions. The tooth is restored by using the Gradia® Direct Flo and Gradia® Direct Posterior composite resin (GC Europe N.V., Leuven, Belgium) in accordance with the manufacturer’s instructions and if necessary a matrix system (Kerr Restauration, Bioggio, Switzerland). The photo-polymerization steps involve use of a LED curing light (Demi™ Ultra, Kerr Restauration, Bioggio, Switzerland). Then, a bite-wing radiograph is taken as a control.

Various strategies are implemented to improve adherence to intervention protocols: the investigator must submit the fluorescence image obtained after caries removal as well as the control radiograph to the randomization website. He must also indicate the adhesive applied.

### Outcome measures

Cases are considered successful if each of the following five FDI World Dental Federation (FDI) criteria has a final score ≤ 3: material fracture and retention, marginal adaptation, radiographic examination (including apical pathologies), postoperative sensitivity and tooth vitality, and recurrence of carious lesions [48, 49]. A score of 4 or 5 indicates that reintervention is necessary and, therefore, the case is classified as a failure in the context of the study. Criteria are scored at 1, 2, and 3 years’ postintervention by two blinded independent trained evaluators. Study assessing the agreement between evaluators for FDI criteria is ongoing. In cases of emergency visits, FDI criteria will be scored by the investigator.

For the primary objective (P versus C excavation) and the main secondary objective (AA versus TA), the outcome will be the success (yes/no) at 1 year, as defined previously.

For the first secondary objective (identification of factors predicting success at 1 and 3 years), the following candidate factors will be investigated: postoperative pain, patient age, type of carious lesion (primary or secondary), location of the carious lesion, and pulp exposure.

For the second secondary objective (description of the consequences of treatment failure and side effects per treatment arm), the following events will be considered: total number of needed visits, number of endodontic treatments, and number of avulsions.

### Data collection methods

Trial data are collected in an eCRF. To promote data quality, investigators, and evaluators (examiners), who will assign scores, are trained in the FDI criteria by means of the e-calib web-based software (www.e-calib.info) and group training sessions. Two independent evaluators will perform the follow-up examinations. If disagreements occur during the evaluations, evaluators will have to reach consensus. Data collection forms can be found in the trial protocol as well as on the randomization website. To promote participant retention and complete follow-up, each patient will receive 90€ on completion of the trial as compensation for attending the follow-up appointments.

### Data management

Data are entered by investigators and evaluators on the eCRF. Fields include range checks for data values. Fields cannot be left blank.

Interactive data controls will be applied for value ranges and presence of and between-form coherence. Data will be kept anonymous, with high-level security storage, with encryption of all data transfers, in compliance with French regulatory and European Clinical Research Infrastructure Network (ECRIN) requirements.

### Statistical methods

The statistical unit for analysis will be the tooth. Only one tooth per patient will be included and treated according to the protocol. The demographic and clinical characteristics of patients and treated teeth will be described for both treatment arms with mean and SD or median and interquartile ranges for quantitative variables and number of subjects, and percentages for qualitative variables. The analyses will be performed according to the intention-to-treat principle.

To evaluate the primary objective, the probability of success in arms P and C at 1 year will be calculated and compared by Fisher’s exact test. The probability of success for each arm (0 for treatment failure and 1 for success, at 1 year) will be assessed by a hierarchical logistic model. This model accounts for the correlation related to the hierarchical structure of the data (several patients treated by one practitioner) as well as any possible inter-practitioner heterogeneity. The model will be adjusted on the type of adhesive (arm AA or TA) and on prognostic factors accounted for in the minimization scheme. If necessary, other adjustment factors might also be considered. In cases of patient non-compliance with follow-up visits, the last investigator’s assessment will be used in the analysis, if there is one.

All analyses related to the primary objective (Fisher’s exact test and the modeling approach) will initially include all patients with an available primary outcome as modified ITT. The number of patients to be included has been increased to account for missing outcomes. Then, three sensitivity analyses will be performed: the first according to the maximal bias approach (missing data considered as “success” in arm C and as “failure” in arm P), the second considering all missing data as successes, and the third considering all missing data as failures.

For the secondary objectives comparing efficacy (as defined for the primary objective) at 1, 2, and 3 years and between arm AA and arm TA at the three different times, similar analyses to those used for the primary objective will be performed. The probability of success for each criterion will be assessed and compared between arms by using the hierarchical logistic model described previously.

Candidate factors for subgroup analyses are patient age, type of carious lesion (primary or secondary), caries risk, lesion location, and pulp exposure. Subgroup analyses will be performed globally, then stratified by type of removal (C and P) and adhesive (AA and TA). In the model analysis, the interaction between a specific factor and the type of removal (arms C versus P) or the adhesive used (arms AA versus TA) will also be considered by its clinical relevance and in the descriptive analyses. The consequences of failure and side effects between arms will be assessed by a descriptive approach (range, median, and quartiles for quantitative variables, number and percentage for qualitative variables).

### Data monitoring, harms and auditing

The data will be monitored by an independent clinical research assistant, who will compare the data entered in the eCRF with those in the patient’s paper clinical record. In case of disagreement, the patient’s investigator will be asked to clarify the data. No interim analysis (other than that described above) is planned. If possible, the follow-up duration will be extended. Concerning harm monitoring, specific adverse events forms can be accessed in the eCRF. Trial management may be audited by the French Department of Health at any time; the audit would be independent of investigators and the sponsor. Investigators will not have access to the final trial data set; the latter will be accessed by clinical research assistants, data managers, and statisticians only.

### Ethical consideration

The Human Research Ethics Committee of the University Hospital of Lyon (Comité de protection des personnes or CPP Sud-Est IV) has approved the study protocol (2014-A00907-40). The protocol is registered with the Agence Nationale pour la Sécurité du Médicament et des Produits de Santé (ANSM, the French National Agency for Medicines and Health Products Safety; no. 2014-A00907-40) and ClinicalTrials.gov (no. NCT02286388). All amendments to the protocol will be justified, submitted to the scientific board, accepted by the CPP Sud-Est IV and recorded by the ANSM. Changes and amendments will be also recorded at ClinicalTrials.gov. Informed consent will be obtained from trial participants or authorized surrogates (parents of children aged 8–18) after trial explanation by an investigator or investigator of the corresponding center. Patients are informed that they have the right to withdraw from the study at any time without giving reasons. Regardless of withdrawal, patients will be provided any treatment in their best interest. Withdrawal will be documented. Data confidentiality was audited by the Comité National Informatique et Liberté (CNIL, National Committee of Informatics and Freedom); last and first names of included patients are not recorded in the database.

No Data Monitoring Committee is needed for a DEep CAries Treatment (DECAT) study as no or very few serious events are expected from this study limited to common dental care. No interim analyses are planned.

### Dissemination of results

The Consolidated Standards of Reporting Trials (CONSORT) guidelines will be used to report the results of this study and the results will be published in international peer-reviewed journals [50]. Authors of the publications will be people involved in the elaboration of protocol, the implementation and conduct of the trial, and the writing of the manuscript and report. The results related to the main objective will be authored by the coordinator, the methodologists, the investigators who have included at least 15 cases and other people who will have significantly contributed to the planning of the trial, its implementation, or the writing of the report.

A summary of the study results will be posted at ClinicalTrials.gov to allow general access to the findings.

Data sharing will be at the participant level. Access to the full protocol can be granted to anyone upon request.

The database will be open after the main analysis to the academic community including meta-analyses on individual data

## Discussion

An alternative to the usual care for caries lesion removal would be more widely adopted by both clinicians and policy makers if the evidence showed that the more conservative partial excavation technique was at least equally successful, less time-consuming, and more cost-effective than the complete excavation procedure. However, a high level of evidence is lacking [18, 19]. The systematic review by Bergenholtz et al., in 2013, found that only a few studies have examined partial caries removal and that the sample size was often fewer than 100 patients [18]. This trial may help address this issue well because it involves 13 centers nationwide, and the recruitment of a minimum of 464 patients should be possible. Two private dental practices are also involved because the patient profiles in public consultation may differ from those in private dental practices. In addition, the inclusion criteria are broad, so patients included are varied, especially in terms of individual caries risk. Hence, the external validity of the data should be optimized.

In addition, this study will provide new insights into the use of antibacterial adhesive systems. Currently, to our knowledge, only one antibacterial adhesive system is available on the professional market (Clearfil™ SE Protect). The adhesive selected as the reference in this trial is similar to Clearfil™ SE Protect except for the antibacterial component to allow evaluating the interest of adding antibacterial components to adhesive resins.

All current recommendations (the SPIRIT statement) were taken into account for the design of the present clinical trial [33]. A complete factorial design would have allowed for exploring the interaction between the management strategies (P versus C excavation, and AA versus TA). However, the impossibility of randomizing the adhesive with endodontic treatment, expected to be more frequent with C excavation, led us to adopt a nested design with two distinct and successive randomizations. The tooth was chosen as the statistical unit, with only one tooth per patient, to ensure the independence between statistical units. If a split-mouth design had been chosen, finding patients with two cavities similar in depth would have been difficult. In terms of internal validity, the sources of bias are limited by the use of centralized randomization (selection bias), strict prospective data record and monitoring (information bias), and blinded patients and evaluators (performance and detection bias). However, because of the nature of the investigation, the investigators cannot be blinded.

The choice of assessment criteria is based on the 16 criteria for evaluating direct restorations published and approved by the FDI [48]. As suggested by Hickel et al., a selection of criteria was chosen to match the objectives of the study [49]. The focus was functional properties (material fracture and retention, marginal adaptation, radiographic examination) and biological properties (postoperative pain and tooth vitality, recurrence of carious lesions).

The clinical procedure is carried out under optimal conditions. One of the main points is that partial removal involves only the tissues close to the dental pulp. Indeed, the cavity margins should include no carious tissue so as to avoid the recurrence of the lesion at the tooth-restoration interface. Decay can reach different surfaces of the tooth, but the restoration must be single and unique to avoid any confusion during the evaluation step. For the same reason, the included tooth must be free of cervical carious lesion.

The present research focuses on indications and outcomes of interventions including the use of dental materials. The availability of new data should help educate decision makers in the dental profession and play a major role in planning evidence-based dental treatment. The results will allow for advancement toward minimum intervention in general as well as minimally invasive dentistry, beneficial for the patient, the practitioner, and the public health care system.

## Trial status

The trial is currently in the recruitment phase.

## Abbreviations

AA, antibacterial adhesive; ANSM, Agence Nationale pour la Sécurité du médicament et des Produits de Santé (French National Agency for Medicines and Health Products Safety); C, complete excavation; CNIL, Comité National Informatique et Liberté (National Committee of Informatics and Freedom); CONSORT, Consolidated Standards of Reporting Trials; CPP, Comité de Protection des Personnes (Ethics Committee); DECAT, DEep CAries Treatment; DGOS, Direction Générale de l’Offre de Soins (Directorate General of Health); eCRF, electronic case report; ECRIN, European Clinical Research Infrastructure Network; FDI, FDI World Dental Federation; P, partial excavation; PHRC, Projet Hospitalier de Recherche Clinique (Hospital Clinical Research Program); SPIRIT, Standard Protocol Items: Recommendations for Interventional Trials, TA, two-step self-etch adhesive

## Additional file

Additional file 1: SPIRIT checklist of the trial. (DOC 133 KB)
